# Improvement of betulinic acid biosynthesis in yeast employing multiple strategies

**DOI:** 10.1186/s12896-016-0290-9

**Published:** 2016-08-17

**Authors:** Chen Zhou, Jing Li, Changfu Li, Yansheng Zhang

**Affiliations:** 1 CAS Key Laboratory of Plant Germplasm Enhancement and Specialty Agriculture, Wuhan Botanical Garden, Chinese Academy of Sciences, Wuhan, 430074 China; 2 University of Chinese Academy of Sciences, Beijing, 100049 China

**Keywords:** Betulinic acid, Betulin, Biosynthesis, *Saccharomyces cerevisiae*

## Abstract

**Background:**

Betulinic acid (BA) is a lupane-type triterpene which has been considered as a promising agent to cure melanoma with no side effects. Considering that BA is naturally produced in small quantities in plants, we previously reported the success in engineering its production in yeast. In the present study, we attempted to improve BA biosynthesis in yeast by the use of different strategies.

**Results:**

We first isolated a gene encoding a lupeol C-28 oxidase (LO) from *Betula platyphylla* (designated as *BPLO*). BPLO showed a higher activity in BA biosynthesis compared to the previously reported LOs. In addition, two yeast platforms were compared for engineering the production of BA, which demonstrated that the WAT11 strain was better to host BA pathway than the CEN.PK strain. Based on the WAT11-chassiss, the Gal80p mutant was further constructed. The mutant produced 0.16 mg/L/OD_600_ of BA, which was 2.2 fold of that produced by the wild type strain (0.07 mg/L/OD_600_).

**Conclusions:**

This study reported our efforts to improve BA production in yeast employing multiple strategies, which included the identification of a novel LO enzyme with a higher activity in BA biosynthesis, the evaluation of two yeast strains for hosting the BA pathway, and the up-regulation of the expression of the BA pathway genes by managing yeast GAL gene regulon circuit.

**Electronic supplementary material:**

The online version of this article (doi:10.1186/s12896-016-0290-9) contains supplementary material, which is available to authorized users.

## Background

Betulinic acid (BA) belongs to lupane-type triterpenes and its derivatives show a wide range of health benefits, such as anti-cancer [1], anti-HIV [2], anti-virus [3], and anti-malarial activities [4]. In particular, BA shows a selectively inhibitory activity against melanoma cells with no side effects on regular ones [5]. Despite these promising activities, commercial application of this compound is limited largely due to its short supply from nature. BA accumulates at low quantities in many plants including *Syzygium jambos* [6], *Ziziphus jujube* [7], *Diospyros kaki* Thunb [8], and *Betula platyphylla* [9]. Of these BA-producing plant species, *Betula platyphylla* is the major source of BA for the drug market. The production of BA has heavily relied on the phytochemical extraction from the *Betula platyphylla* barks. In China, around 230,000 t of the *Betula platyphylla* barks per year are consumed for the extraction of BA (this statistics data was retrieved from an annual report from the national statistics department of China). Apparently, the overexploitation on the *Betula platyphylla* barks would be problematic for continually supplying BA in a long term aspect.

The biosynthetic pathway of BA has been elucidated before [10, 11]. Cyclization of 2,3-oxidosqualene by lupeol synthase to produce lupeol is the initial committed step toward BA biosynthesis, and the lupeol synthase gene from *Arabidopsis thaliana* (*AtLup1*) has been isolated [12]. Lupeol is then successively oxidized at its C28 position to yield BA by a cytochrome P450 enzyme, lupeol C-28 oxidase (LO). We previously reported that an amyrin oxidase from *Catharanthus roseus* (*CrAO*) showed the LO activity [11]. By the combinatory expression of *CrAO* and *AtLup1*, we succeeded in engineering the BA production in *S. cerevisiae* via the yeast endogenous 2,3-oxidosqualene [10, 11, 13]. In addition to *CrAO* from *Catharanthus roseus*, the genes encoding other LO enzymes had been isolated from several plant species, including *CYP716A12* from *Medicago truncatula* and *CYP716A15* from *Vitis vinifera* [14]. Despite that *Betula platyphylla* bark is the major source for the production of BA, the LO gene of this plant remains not isolated. Therefore, here we reported on the isolation and characterization of the LO gene from *Betula platyphylla* bark (designated as *BPLO*). Relative to the previously identified LOs described above, BPLO showed a higher BA-producing activity in yeast.

After the success of engineering BA biosynthesis in yeast, the next challenge is to boost its production. When foreign pathways are introduced into a microbial host, its existing metabolic network would tightly control carbon fluxes to foreign compounds. The yeast strain that we previously used for engineering the BA production is a laboratory *S. cerevisiae* strain WAT11 [10]. The yeast WAT11 strain has a genomically integrated *Arabidopsis thaliana* NADPH-CYP reductase as a redox partner for the CYPs [15], and is usually used to characterize plant cytochrome P450 enzymes [16, 17]. The *S. cerevisiae* CEN.PK is another yeast strain which has often been employed for synthesizing a wide range of products in industry [18, 19]. The *S. cerevisiae* CEN.PK strain contains a significantly higher content of ergosterol than other *S. cerevisiae* strains [20]. Genomic sequencing revealed that the CEN.PK strain contains a number of SNPs in the coding regions of ergosterol and fatty acid pathway enzymes [21]. The authors suggested that those SNPs might be responsible for the high-level ergosterol accumulation of this strain. Both ergosterol and BA are cyclized from 2,3-oxidosqualene, a common precursor for the biosynthesis of sterols and triterpenes. A higher accumulation of ergosterol in the CEN.PK strain prompted us to test whether it is better than other yeast strains to engineer the BA production. Therefore, in this study, the BA pathway was integrated into the genomes of both the WAT11 and CEN.PK strains and their BA-producing abilities were compared.

In our previous attempts to engineer the BA production in yeast, the BA pathway was expressed in either a constitutive or a galactose-inducible manner, and galactose-inducible promoters (*GAL1* or *GAL10* promoter) appeared to be stronger to direct the expression of the BA pathway genes [10, 13]. Therefore, in this study, all the BA pathway genes were cloned under galactose-inducible promoters. The induction of GAL genes is controlled by a protein complex of Gal3p, Gal4p and Gal80p [22]. Gal4p is a transcriptional activator that binds to the upstream activation sequences of GAL genes to activate the transcriptions of the genes. When glucose is used as the sole carbon source, the activation domain of Gal4p is masked by the repressor Gal80p, thereby preventing the transcriptions of GAL genes. In the presence of galactose, the signal transducer Gal3p binds to Gal80p to expose the activating domain of Gal4p, leading to the activation of GAL genes. It has been reported that a yeast mutant lacking Gal80p produced higher levels of carotenoids when the carotenoid pathway genes were controlled by galactose-inducible promoters [23]. Here, we also reported that the Gal80p mutant increased the BA production, which was up to 2.2-folds of that produced by the wild yeast strain.

## Results

### Isolation and functional analysis of BPLO

A 1082 bp-cDNA fragment was amplified using the degenerate primers 1/2 from the bark of *B. platyphylla* where BA primarily accumulates [9]. The resulting cDNA fragment showed sequence identities to the genes coding for the previously reported LO enzymes, and therefore its full-length cDNA (designated as *BPLO*) was further cloned by RACE-PCR technique using primers 3–6. BPLO was also officially named as CYP716A180 by the standard P450 nomenclature committee [24]. During our biochemical characterization of BPLO, the *BPLO* gene was also isolated by another group from the Northeast Forestry University of China, and was named as *ATH1* (GenBank accession no. KJ452328.1). The predicted protein sequence of BPLO showed 79–81 % identity at the amino acid level with the previously published LOs, including CrAO from *C. roseus*, CYP716A12 from *M. truncatula* and CYP716A15 from *V. vinifera*.

**Fig. 1:**
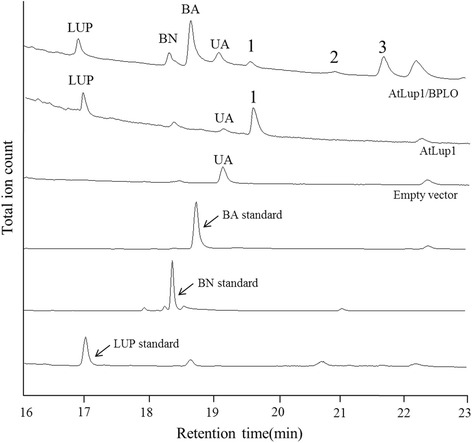
Functional analysis of *BPLO* by its co-expression with *AtLup1* in yeast cells. GC–MS analysis was shown for the products from the yeast cells expressing AtLup1/BPLO yielding lupeol (LUP), betulin (BN), betulinic acid (BA), and the unknown products (peaks 2–3), and the cells expressing *AtLup1* alone yielding lupeol and the unknown peak 1. The identities of LUP, BN and BA were confirmed with their corresponding chemical standards by comparing their mass fragmented products (Additional file 1: Figure S1). The identities of the peaks 1–3 were not determined due to the absence of their chemical standards, and their MS spectrums were shown in Additional file 1: Figure S2

To examine the activity of BPLO, the *BPLO* gene was co-expressed in the WAT11 yeast strain with *AtLup1*. The yeast cells transformed with the empty vector or expressing *AtLup1* alone served as the control. The WAT11 strain contains an *Arabidopsis thaliana* cytochrome P450: NADPH reductase ATR1 [15] that is required for the activity of plant P450 enzymes. Compared to the empty vector control, the expression of *AtLup1* alone led to the production of lupeol and an unknown peak 1. Due to the absence of the authentic standard, we were not able to determine the identity of the peak1, but it was assumed to be 3α,20-dihydroxylupane (lupanediol), as it was previously found as the second major product of the AtLup1, in addition to lupeol [25]. When *AtLup1* and *BPLO* were co-expressed, lupeol was successively oxidized at its C-28 position by BPLO to yield betulin (BN) and betulinic acid (BA) which were not produced by the control yeast cells (Fig. 1). The identities of BN and BA products were confirmed by comparisons with their authentic standards (Additional file 1: Figure S1). These data clearly suggested that BPLO functions as a LO enzyme. Interestingly, BPLO also seemed to be able to convert the peak 1 to the products of peaks 2 and 3 with the peak 2 being negligible (Fig. 1). The peak 2 and peak 3 could be the oxidized alcohol and acid products of lupanediol, respectively, which was presumably catalyzed by BPLO at the C-28 position of lupanediol. The mass spectrums of the trimethylsilyl-derivatives of the peaks 1–3 were shown in Additional file 1: Figure S2.

**Fig. 2:**
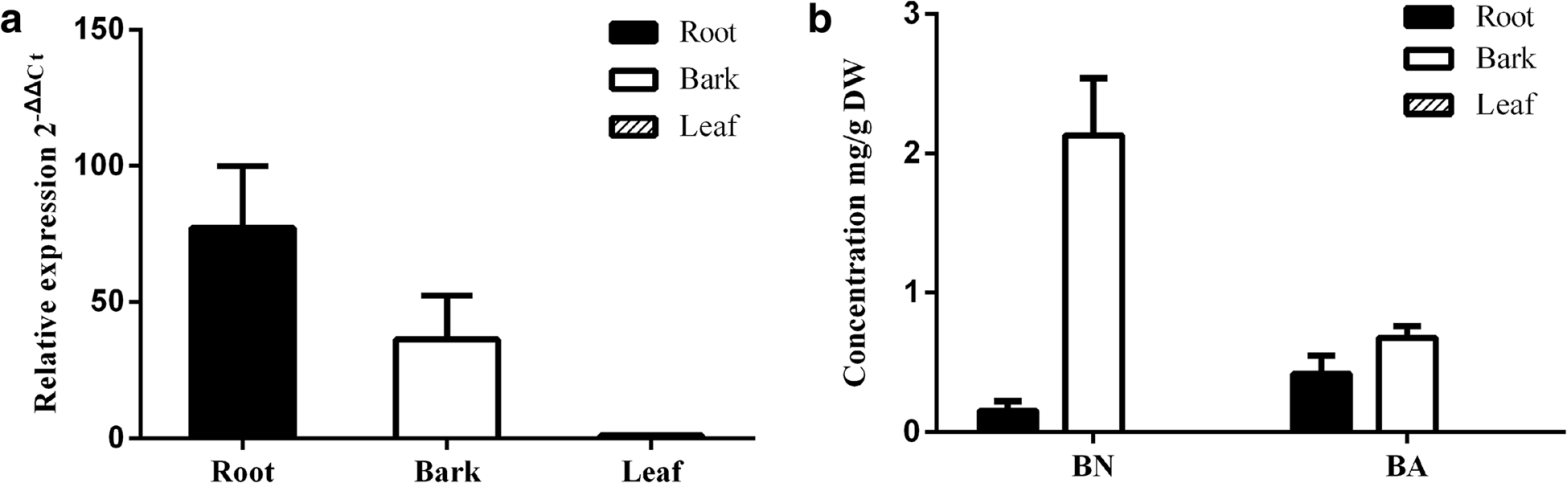
The accumulation pattern of the BPLO transcripts and its enzymatic products in *B. platyphylla* tissues. **a** the BPLO transcripts measured by qRT-PCRs; **b** the BPLO enzymatic products (BN and BA) measured by GC-MS analysis. BN, betulin; BA, betulinic acid. Error bars represent the standard errors (SE) of the means calculated from three biological replicates

To examine the relevance of the *BPLO* transcript to BA biosynthesis in *Betula platyphylla*, we have investigated the accumulation pattern of the *BPLO* transcripts and its enzymatic products (BN and BA) across different tissues of *Betula platyphylla*. The *BPLO* transcript was mostly observed in the roots and barks while absent in the leaves (Fig. 2a). On the other hand, GC-MS analysis showed the accumulation of the BPLO products (BN and BA) in the roots and barks, whereas none of them was detected in the leaves (Fig. 2b). Therefore, the *BPLO* gene expression pattern is overall consistent with the BA accumulation in vivo. However, a higher level of the *BPLO* transcript was detected in the roots relative to the barks whereas the barks contain higher levels of the BPLO products than the roots. This inconsistence may be caused by the transport of BPLO products from the roots to the barks or less precursor lupeol in the roots supplied for BPLO. We also examined the level of lupeol in these tissues, but it was not detectable probably due to its complete conversion to BN and BA.

### BPLO showed a higher BA-producing activity than the previously reported LOs

**Fig. 3:**
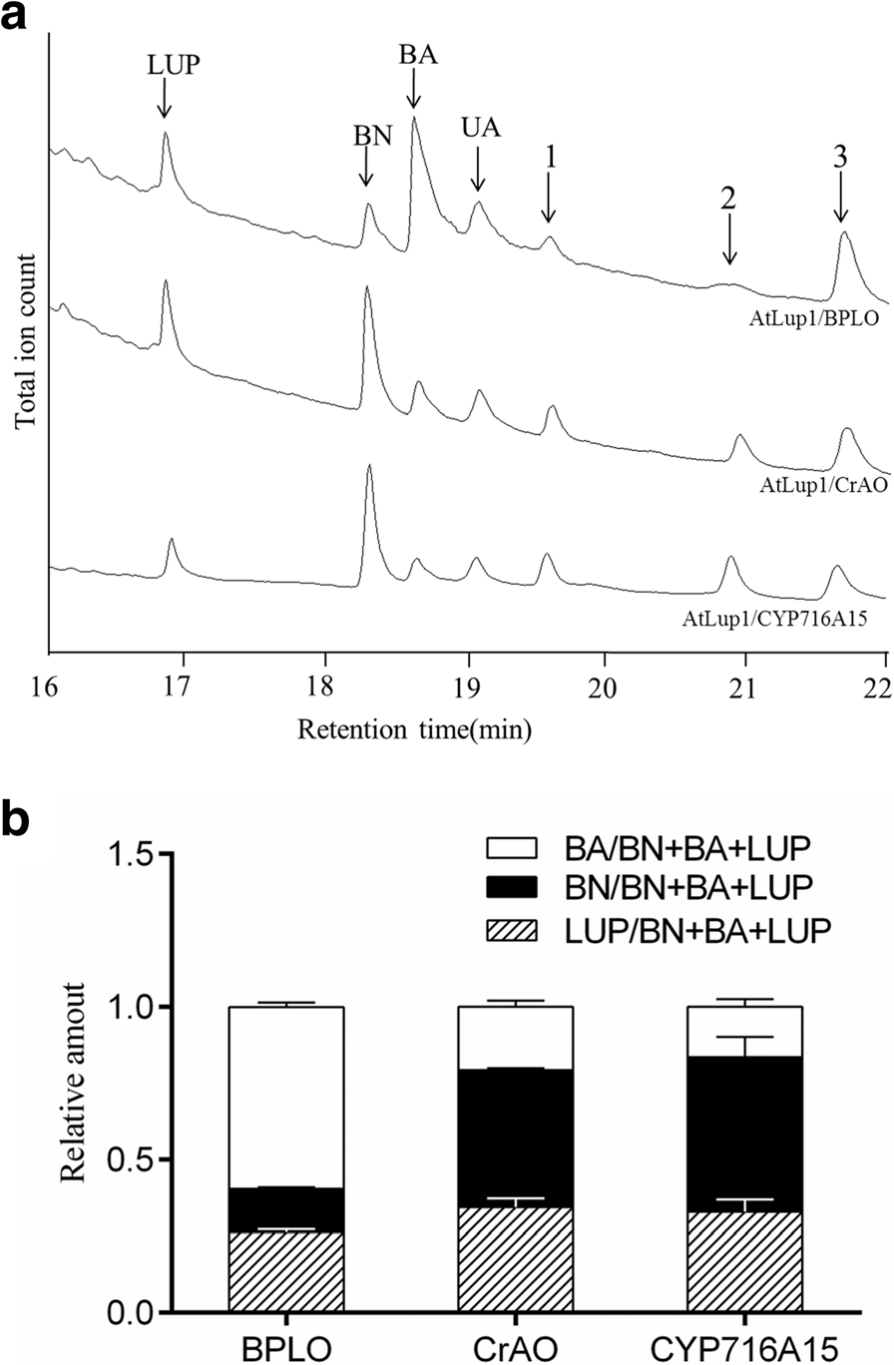
Comparison of the BA-producing activities between BPLO and the previously reported LOs (CrAO and CYP716A15) in yeast. The LO genes were individually co-expressed with *AtLup1* in the WAT11 yeast strain. **a** Total ion chromatograms were shown for the products by expressing BPLO, CrAO and CYP716A15; **b** the relative amounts of the products lupeol (LUP), betulin (BN), and betulinic acid (BA) produced by the transgenic yeast cells expressing the individual LO. “LUP/BN + BA + LUP” represents the ratio of the yield of the total products (LUP, BN and BA) divided by the LUP yield. Similarly, “BN/BN + BA + LUP” and “BA/BN + BA + LUP” represent the ratios of the total product yield divided by the yield of BN and BA, respectively. Error bars represent the standard errors of the means calculated from two biological replicates

To evaluate the BA-producing activity between BPLO, CrAO and CYP716A15, these LOs were initially expressed in the WAT11 strain alone, and each resultant yeast culture was then fed with 50 μM lupeol as the LO substrate. After galactose induction, very low levels of BA were produced in all the three yeast cultures (Additional file 1: Figure S3A), indicating that the substrate lupeol was indeed taken up by the yeast strain but at a very low efficiency. Interestingly, even under this condition, BPLO seemed to produce higher levels of BA than CrAO and CYP716A15 (Additional file 1: Figure S3B). To further compare their activities, the three LO genes were then co-expressed with the lupeol synthesizing gene *AtLup1* in the WAT11 strain. After induction with 2 % galactose for 60 h, the yeast cultures were all acidified to pH 2.0 and extracted with ethyl acetate for GC-MS analysis. Once again, BPLO produced the most BA while the lowest amount of BN in comparisons with the CrAO and CYP716A15 (Fig. 3). Thus, these results strongly suggested that BPLO displayed a higher BA-producing activity than the previously characterized CrAO and CYP716A15. On the other hand, like the results shown in Fig. 1, the unknown products (peaks 1–3) were also observed in this experiment. Interestingly, compared to CrAO and CYP716A15, BPLO resulted in relatively higher levels of the peak 3 while lower amounts of the peak 2 (Fig. 3a), which pattern was very similar to that of these LOs’ activities on the BA pathway. This data may also reflect the truth that BPLO catalyzes a higher C28-oxidizing activity toward lupeol or lupeol-like compounds than CrAO and CYP716A15.

### The WAT11 strain was better than the CEN.PK strain to host BA pathway

**Fig. 4:**
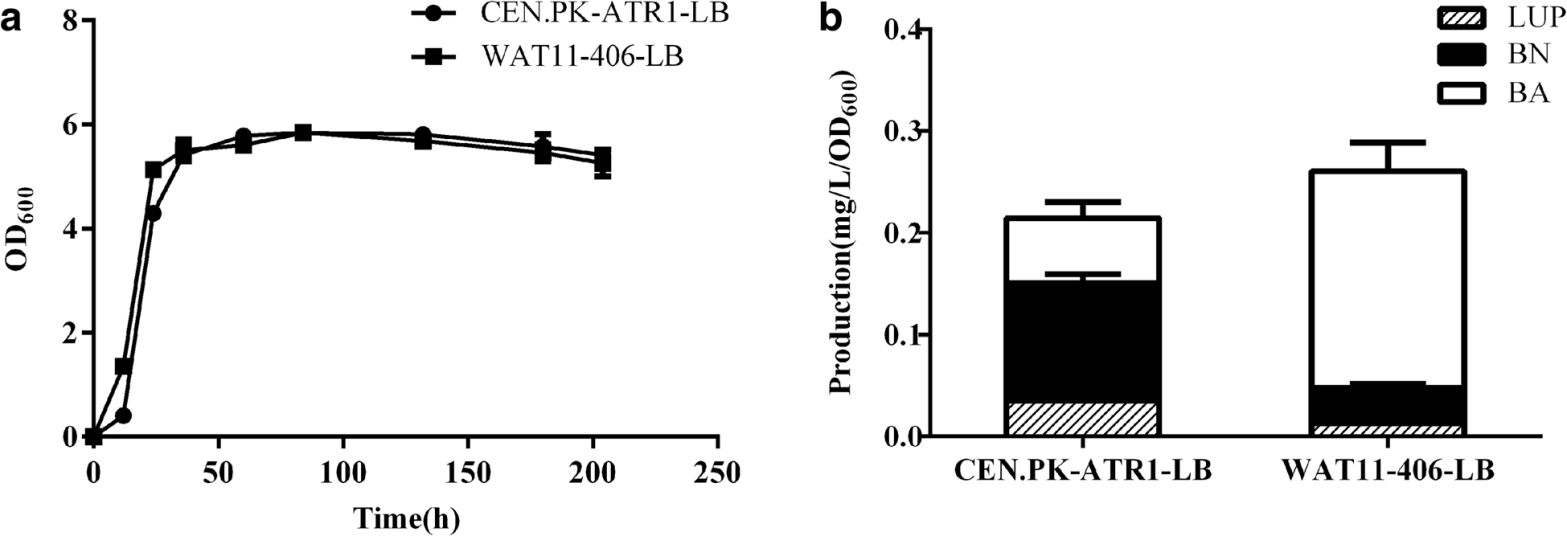
Comparison of CEN.PK and WAT11 strains for their BA-producing abilities. **a** the growth properties of the both strains measured by checking the optical density at the wave length of 600; **b** the BA-producing abilities of the both strains measured by calculating the yields of lupeol (LUP), betulin (BN) and betulinic acid (BA). Error bars represent the standard errors of the means calculated from three biological replicates

The yeast strain CEN.PK was demonstrated to be a triterpene hyper producer [26], which prompted us to expect that the strain CEN. PK would be a better host to engineer the biosynthesis of BA. Thus, the strain CEN.PK was then investigated for its BA-producing ability compared to the WAT11 yeast strain. The WAT11 strain contains the gemonically integrated ATR1 [15] that is required for the activity of BPLO. The exactly same ATR1 expression cassette was amplified from the WAT11 strain and integrated into the chromosome of the CEN.PK strain to generate the strain CEN.PK-ATR1 via an integrative expression vector pRS406. For a fair comparison, the empty vector pRS406 was also integrated into the WAT11 strain to give the strain WAT11-406. The BA pathway was then integrated into both the WAT11-406 and CEN.PK-ATR1 strains, generating the strains of WAT11-406-LB and CEN.PK-ATR1-LB, respectively. The integrated yeast strains were cultured for assessing their BA-producing abilities. There were no significant differences in their growth curves of both strains (Fig. 4a). After the galactose-induction for 9 days, the yeast cells and medium were partitioned by centrifugation and separately extracted with ethyl acetate for GC-MS analysis. The BPLO products (BN and BA) were majorly detected in the medium whereas very low amounts of them accumulated within the cells of both strains (Additional file 1: Figure S4), suggesting that both strains were able to highly transport the products out of cells. For the products in the medium, WAT11 strain accumulated about 3-fold higher BA levels and relatively lower amount of BN compared to the CEN.PK strain (Fig. 4b), suggesting that the WAT11 strain had a more efficient conversion of BN to BA than the CEN.PK strain. On the other hand, compared to the WAT11 strain, a significantly higher level of lupeol accumulated in the CEN.PK strain (Fig. 4b). The total yields of lupeol, BN and BA would reflect the carbon fluxes through the BA pathway. The data of the Fig. 4 indicated that WAT11 strain provided greater metabolic fluxes through the BA pathway than the CEN.PK strain. Thus, further engineering of the BA production of this study was performed based on the WAT11 stain.

### The inactivation of Gal80p improved the BA yield based on the WAT11 strain

**Fig. 5:**
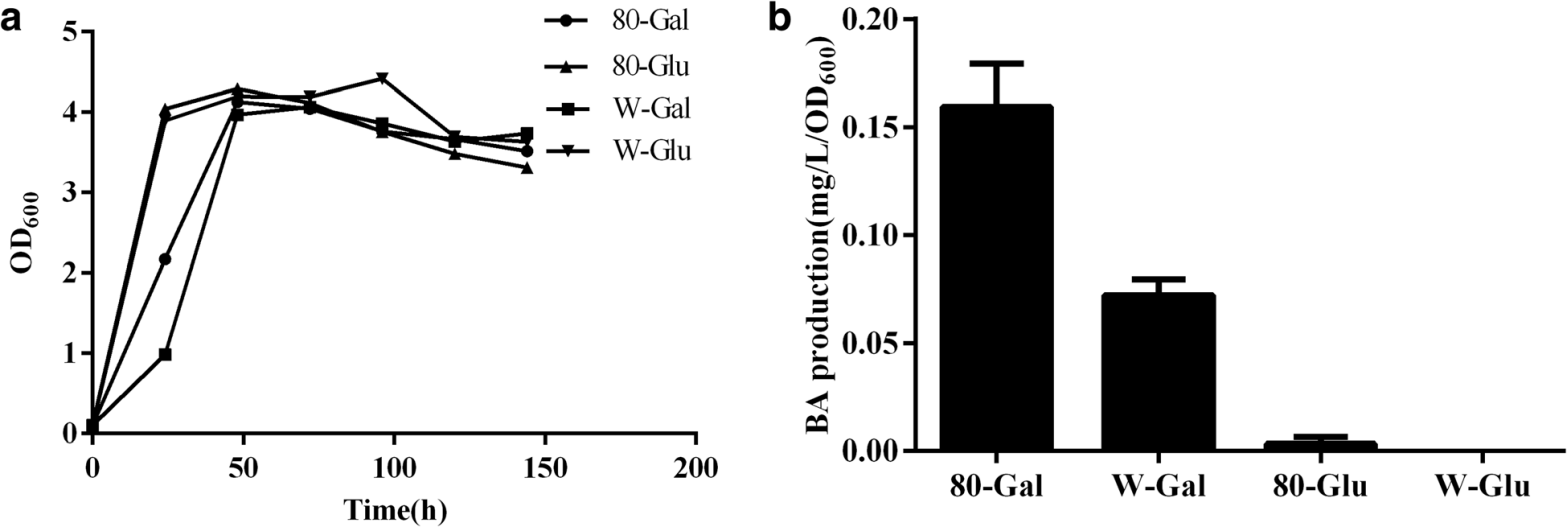
Yeast growth properties **a** and betulinic acid production **b** were compared between the wild type strain (WAT11-LB) and the Gal80p mutant (WAT11-LB-ΔGal80). 80-Gal, the mutant strain cultured under 2 % galactose; W-Gal, the wild type strain cultured under 2 % galactose; 80-Glu, the mutant strain cultured under 2 % glucose; W-Glu, the wild type strain cultured under 2 % glucose. Error bars represent the standard errors of the means calculated from three biological replicates

In this study, the BA pathway genes were all cloned under the control of galactose-inducible promoters and their transcriptions are galactose-inducible. The induction of GAL genes is controlled by the protein complex of Gal3p, Gal4p and Gal80p [22]. The expression level of GAL genes could be increased in the Gal80 mutant when using non-inducing carbon source [27]. It has also been reported that the loss of Gal80p function in yeast improved carotenoid production when the carotenoid biosynthetic pathway genes were cloned under galactose inducible promoters [23]. Based on the BA-producing strain of this study, we also investigated the effect of the Gal80 mutation on the BA production. Using homologous and loxp sites-based specific recombinations, the Gal80p inactivation was made based on the WAT11-LB strain, which was confirmed by genomic DNA PCRs (Additional file 1: Figure S5). The mutant strain was then independently cultured using 2 % glucose or 2 % galactose as the carbon sources. As a control, the corresponding wild type strain WAT11-LB was cultured in the same ways. On 2 % glucose, the growth property of the mutant resembled that of the wild strain (Fig. 5a) and the BA production was hardly detected whether or not the Gal80p was inactivated (Fig. 5b). On 2 % galactose, the BA production from the mutant was 1.2 fold higher than that of the wild strain, suggesting that the Gal80p disruption improved the expressions of the BA pathway genes, which were cloned under the control of galactose inducible promoters. This assumption was proved by the gene expression analysis of the *BPLO* gene from both the mutant and the wild strain by real-time PCRs (Additional file 1: Figure S6).

## Discussion

The success of engineering the BA production in yeast cells has previously been reported by our group [10, 11, 13]. In the present study, we attempted to increase the BA yields in yeast using multiple strategies, which include the identification of a higher BA-producing enzyme, the assessment of two different yeast strains, and the increase of the BA pathway gene transcriptions. The bark of white birch is the major source for the BA production, while the BA-producing enzymes in this species have never been reported before this study. Using a homology-based strategy, we have successfully isolated the gene encoding a novel LO enzyme (BPLO) from white birch bark. Compared with the previously reported LOs, BPLO produced the most BA while the lowest BN (Fig. 3), suggesting that BPLO exhibits a higher activity of converting BN to BA than the other LOs.

Previously, the *S. cerevisiae* CEN.PK strain was revealed to contain higher levels of ergosterol than other *S. cerevisiae* strains [20]. We also observed that the concentration of ergosterol in CEN.PK strain is higher than that in the WAT11 strain (data not shown). Ergosterol is derived from 2,3-oxidosqualene which is also a precursor for the biosynthesis of BA, thus, we initially anticipated that the CEN.PK strain might be a better strain for the BA-production. However, the comparison of the yield of the total products (LUP, BN and BA) between both strains (Fig. 4b) indicated that the WAT11 strain had greater metabolic fluxes through the BA-pathway than the CEN.PK strain. Moreover, the ratios between the individual products suggested that the WAT11 strain was more efficient than the strain CEN.PK in the conversion of BN to BA (Fig. 4b). Given the relatively comparable cell densities of both strains (Fig. 4a), it appeared that the better strain for engineering the BA-production is the WAT11 rather than the CEN.PK. Both strains differ in many respects, the mechanism by which the WAT11 strain performed better for the BA-production is not clear.

In the current study, the BA pathway genes were cloned under the control of galactose inducible promoters. It is well accepted that a protein complex of Gal3p, Gal4p and Gal80p involve in managing the GAL gene transcriptions, and of which the Gal80p acts as an inhibitor in transcribing the GAL genes [22]. On the basis of the WAT11-LB strain which bears the BA-pathway, the disruption of the Gal80p gene caused a higher transcript level of the *BPLO* gene on 2 % galactose (Additional file 1: Figure S6), which ultimately increased the BA levels (Fig. 5). When the Gal80p was inactivated, its inhibiting effects on galactose-dependent gene inductions might be alleviated, resulting in relatively higher transcriptions of the BA pathway genes in the mutant than those in the wild strain. However, when 2 % glucose was used as the sole carbon source, the BA production was inhibited, and this inhibition seemed not to be unlocked upon the Gal80p disruption (Fig. 5b). This data is reasonable because that the GAL gene regulation via the Gal80p depends on the presence of galactose while not glucose [23].

## Conclusions

The lupeol C-28 oxidase from *B. platyphylla* (BPLO) exhibited higher activities in BA biosynthesis, comparing to the previously reported LOs. The yeast strain WAT11 strain was better to host BA biosynthesis than the CEN.PK strain. Based on the BA-producing WAT11 strain, the Gal80p mutant improved the BA biosynthesis up to 2.2 folds relative to the wild type strain.

## Methods

### BPLO cDNA isolation and its gene expression analysis

All the primers used in this study were shown in Additional file 1: Table S1. For the homology-based cloning of *B. platyphylla* lupeol C-28 oxidase cDNAs, degenerate primers 1/2 were designed based on the highly conserved domains of the LOs including CYP716A15 from *V. vinifera* (GenBank accession no. AB619802.1), CYP716A12 from *M. truncatula* (GenBank accession no. FN995113.1) and CrAO from *C. roseus* (GenBank accession no.JN565975). RT-PCRs were performed with the cDNA template that was prepared from the bark of 4 month-old *B. platyphylla* seedling*.* A 1082-bp amplified product was obtained and cloned into pMD18-T vector for sequencing. Based on DNA sequence analysis, the amplified product was found to show about 80 % amino acid identity to the previously identified LOs [11, 14], indicating that the gene product may code for the *B. platyphylla* LO (designated as *BPLO*). The full length cDNA of *BPLO* was then recovered by RACE-PCRs using primers 3–6. To examine the *BPLO* gene expression pattern in *B. platyphylla*, cDNA was synthesized from equal amounts of total RNAs that were extracted from its different tissues. The *B. platyphylla* actin gene (GenBank accession no. EU588981.1) was chosen as an internal standard to normalize the variation of the cDNA preparations. For each sample, qRT-PCR was performed with four technical replicates on three biological replicates using FastStart Universal SYBR Green Master (Roche, Mannheim, Germany). The *BPLO* transcript was amplified with primers 7/8 while the primers 9/10 were used for amplifying the *B. platyphylla* actin gene. The thermal cycling conditions were set as follows: 95 °C for 10 min, followed by 40 cycles of 95 °C for 15 s, and 60 °C for 60 s.

### Functional analysis of BPLO in comparisons with the previously published LOs

The Open Reading Frame (ORF) of *BPLO* was amplified with primers 11/12 from the birch barks by RT-PCRs. Similarly, the ORFs of other LO cDNAs, including *CYP716A15* from *V. vinifera* and* CrAO* from *C. roseus*, were amplified from their host plant leaves using primers 13–16. The ORFs of these LO cDNAs were then cloned into a yeast expression vector pESC-TRP by standard enzyme digestions and ligations. For in vivo functional analysis, these plasmids were co-transformed into *S. cerevisiae* WAT11 strain [15] with the plasmid pESC-HIS-AtLup1, which gives rise to the supply of lupeol [12]. These transgenic yeast strains were initially grown up at 30 °C in an appropriate SD drop out medium with 2 % (w/v) glucose. Then yeast cultures were collected by centrifugation, washed three times in sterile water, and re-suspended to an OD_600_ of 0.8 in 30 mL of SD dropout medium containing 2 % (w/v) galactose. After 60 h of the induction, the growth medium were collected by centrifugation, all acidified to pH 2.0 using 2 M HCl, and extracted with ethyl acetate. The ethyl acetate fractions were dried by a rotary evaporator and derivatized using BSTFA at 80 °C for 30 min prior to GC-MS analysis. For the extraction from the cell pellets, yeast cells were washed with 0.1 mM Tris-HCl buffer (pH 9.0), re-suspended in 0.5 ml of lysis buffer consisting of 50 mM potassium phosphate (pH 7.0), and broken by glass beads. The cell-free extracts were then acidified to pH 2.0 and extracted with ethyl acetate for GC-MS analysis as described above. The contents of lupeol, BN and BA were measured using standard curves.

### Assessments of the WAT11 and CEN.PK yeast strains for their BA-producing abilities

For this experiment, BPLO was chosen to reconstitute the BA pathway as it exhibited a higher BA-producing activity in comparisons with the other LOs, which was concluded from the functional analysis described above. To compare the *S. cerevisiae* WAT11 strain and CEN.PK strain for their BA-producing abilities, the BA pathway genes (*BPLO* and *AtLup1*) were expressed in both strains in an integrative manner. To construct the integrative expression vectors, the BA expression cassette “Gal1 promoter-AtLup1-CYC1 terminator-Gal10 promoter-BPLO-ADH1 terminator” was amplified from the plasmid pESC-TRP-AtLup1-BPLO with primers 17/18 and cloned into a yeast integration expression vector YIplac204 under *Pst*I and *Xba*I sites, yielding the construct YIplac204-AtLup1-BPLO. The expression cassette of the plant cytochrome P450 reductase, “Gal10-CYC1 promoter-ATR1-CPR1 terminator”, was amplified from the WAT11 genomic DNA [15] using primers 19/20 and inserted into a yeast integration expression vector pRS406 to give the construct pRS406-ATR1. For the integrative expression in the CEN.PK strain, the construct pRS406-ATR1 was linearized by *Stu*I digestion and the linear DNA molecule was then transformed into the CEN.PK strain to give the strain CEN.PK-ATR1. Based on the strain CEN.PK-ATR1, the linearized construct YIplac204-AtLup1-BPLO by *EcoRV* digestion was transformed to generate the strain CEN.PK-ATR1-LB. For the integration expression in the WAT11 strain, the empty vector pRS406 was first linearized with *Stu*I and transformed into the WAT11 strain to give the strain WAT11-406. The construct YIplac204-AtLup1-BPLO was then linearized with *EcoRV* and transformed into the WAT11-406 strain to give the strain WAT11-406-LB. Those integrated yeast strains were grown in SD-Ura-Trp-double dropout liquid medium. Relevant metabolites including lupeol, BN and BA were extracted and analyzed by GC-MS as described above, and their concentrations were quantified using ursolic acid as an internal standard.

### Creation of GAL80p mutant based on the strain WAT11-LB strain

The selection marker “LoxP-kanMX-loxP” was amplified from the plasmid pUG6 [28] using primers 21/22. A 393-bp of 5’-Gal80p target and 393-bp of 3’-Gal80p target were amplified from the WAT11 strain genomic DNA using primers 23–26. The two flanking targets were then linked to the selection marker “LoxP-kanMX-loxP” by overlapping PCRs with primers 23/24 to form the Gal80p disruption cassette, in which the selection marker was positioned between the two targets. The correctness of the Gal80p disruption cassette was confirmed by cloning it into the vector pMD18-T for sequencing. After digestions with *BamH*I and *EcoR*I from the T-vector, the linear Gal80p disruption cassette was gel-purified and transformed into the strain WAT11-LB. After selections with 200 mg/L G418, stable transformants containing the disruption cassette were obtained. The selection marker was then removed by a recombinase which was expressed by transforming the recombinase expression vector pYPT2 [29] into the transformants. Finally, the repressor Gal80p was successfully disrupted, yielding the strain WAT11-LB-△Gal80.

### Metabolite extraction from *B. platyphylla* tissues

To examine the BA accumulation pattern in *B. platyphylla* tissues, 0.1 g of dried plant materials (roots, barks and leaves) were extracted with 5 mL of 95 % ethanol for 30 min. The total extract was centrifuged and the pellet was re-extracted twice with 95 % ethanol. The combined supernatants were evaporated and re-dissolved in 2 mL of methanol for GC-MS analysis.

### GC-MS analysis

GC-MS analysis was performed in an Agilent 7890A GC machine (Agilent Technologies, USA) as described previously [11]. Ursolic acid (UA) was used as an internal standard to quantify the BA production. Compounds were identified by comparing their retention times and mass spectrums with those of corresponding authentic standards.

## Additional file

Additional file 1: Table S1. Primers used in this study. **Figure S1.** The mass fragmented patterns of the products lupeol (LUP), betulin (BN) and betulinic acid (BA) as well as their corresponding chemical standards. **Figure S2.** The mass spectrums of the unknown peaks 1–3 shown in the Fig. 1 of the main text. **Figure S3.** GC-MS analysis of the products extracted from the lupeol C28-oxidase (LO) alone-expressed yeast cultures fed with lupeol. **Figure S4.** The production of betulinic acid (BA) was mostly found in the yeast culture mediums of both CEN.PK-ATR1-LB and WAT11-406-LB strains with relatively much less BA being detected inside the cells. **Figure S5.** Confirmation of the Gal80p gene disruption by diagnostic PCRs. **Figure S6.** Comparison of the *BPLO* transcripts between the wild type strain WAT11-LB and the mutant WAT11-LB-△Gal80 under 2 % galactose as the carbon source. (DOCX 831 kb)
