# Supplementary material for: Improvement of betulinic acid biosynthesis in yeast employing multiple strategies
Source: BMC Biotechnol. 2016 Aug 17;16:59. doi: 10.1186/s12896-016-0290-9 (PMC4989488; doi:10.1186/s12896-016-0290-9)
Supplement: Additional file 1: Table S1. — Primers used in this study. Figure S1. The mass fragmented patterns of the products lupeol (LUP), betulin (BN) and betulinic acid (BA) as well as their corresponding chemical standards. Figure S2. The mass spectrums of the unknown peaks 1–3 shown in the Fig. 1 of the main text. Figure S3. GC-MS analysis of the products extracted from the lupeol C28-oxidase (LO) alone-expressed yeast cultures fed with lupeol. Figure S4. The production of betulinic acid (BA) was mostly found in the yeast culture mediums of both CEN.PK-ATR1-LB and WAT11-406-LB strains with relatively much less BA being detected inside the cells. Figure S5. Confirmation of the Gal80p gene disruption by diagnostic PCRs. Figure S6. Comparison of the BPLO transcripts between the wild type strain WAT11-LB and the mutant WAT11-LB-△Gal80 under 2 % galactose as the carbon source. (DOCX 831 kb) [file 12896_2016_290_MOESM1_ESM.docx]

Additional file1

Table S1. Primers used in this study^a^

| No | Name | Sequence (5’-3’) | Description |
| --- | --- | --- | --- |
| 1 | Pf1 | RTTYTCMAAYGARAAYAAACTTGTT | degenerate primer used for cloning *BPLO* |
| 2 | Pr1 | AAWGTRTAWGGAGCWGGYCC | degenerate primer used for cloning *BPLO* |
| 3 | 5’RACE-F | CTTAAGGAACTGGGGAAGCAAC | for cloning *BPLO* in 5’RACE-PCRs |
| 4 | 5’RACE-R | GTCCGGCCACCAGGCAGTTA | for cloning *BPLO* in 5’RACE-PCRs |
| 5 | 3’RACE-F | GGAGTGCGGAATACTTCCCAGA | for cloning *BPLO* in 3’RACE-PCRs |
| 6 | 3’RACE-R | CCGAGTAGATTTGAAGGAAGGG | for cloning *BPLO* in 3’RACE-PCRs |
| 7 | BPLO-RT-F | AGTAGATTTGAAGGAAGGGGAC | for amplifying *BPLO* transcripts in qRT-PCRs |
| 8 | BPLO-RT-R | TGAGGGTAAAGGCGAACGGG | for amplifying *BPLO* transcripts in qRT-PCRs |
| 9 | Be-Actin-F | CATCTCTGATCGGAATGGAAG | for amplifying actin transcripts in qRT-PCRs |
| 10 | Be-Actin-R | AGATCCTTTCTGATATCCACG | for amplifying actin transcripts in qRT-PCRs |
| 11 | BFF | CGCGAATTCATGGAGCATTTCTACCTC | for the expression of BPLO in yeast cells |
| 12 | BFR | GAAGCGGCCGCTCACGCTTTGTGAGGGTA | for the expression of BPLO in yeast cells |
| 13 | V15F | CTTGCGGCCGCATGGAGGTGTTCTTCCTC | for the expression of CYP716A15 in yeast cells |
| 14 | V15R | ACCAGATCTCTATGGTTTGTGAGGATG | for the expression of CYP716A15 in yeast cells |
| 15 | CF | GGCGGATCCATGGAGATCTTCTATGTC | for the expression of CrAO in yeast cells |
| 16 | CR | CGCGGTACCTTATGCATTAATGTGAGG | for the expression of CrAO in yeast cells |
| 17 | YIP-F | TCTCTGCAGGAGCGACCTCATGCTATACCTG | for amplifying the betulinic acid expression cassette |
| 18 | YIP-R | TCTTCTAGACTTCGAGCGTCCCAAAACCT | for amplifying the betulinic acid expression cassette |
| 19 | P-ATR1-SalI | GTCGACCTTCAATTTAATTATATCAG | for amplifying the ATR1 expression cassette |
| 20 | T-ATR1-ClaI | ATCGATGTATGTTGTCTTTGAAGATGCA | for amplifying the ATR1 expression cassette |
| 21 | PUG6-KAN-F | cgaccagcgtatacaatctcgatagttggtttcccgttctttccactcccgtc AGGTCGACAACCCTTAATAT | for amplifying“LoxP-*kanMX*-loxP” |
| 22 | PUG6-KAN-R | ttcgtttttataacgttcgctgcactgggggccaagcacagggcaagatgctt TATAGGGAGACCGGCAGATC | for amplifying“LoxP-*kanMX*-loxP” |
| 23 | 80UP-F | GGATCCCCAATGCTAATCCGGTCACTG | for amplifying 5'-Gal 80 sequence |
| 24 | 80DOWN-R | GAATTCATCAGTTTTTGAAGGCAGCCT | for amplifying 3'-Gal 80 sequence |
| 25 | 80DOWN-5 | GCCCTGTGCTTGGCCCCCAGTGCAGCGAACGTTATAAAAACGAA | for amplifying 3'-Gal 80 sequence |
| 26 | 80UP-3 | GACGGGAGTGGAAAGAACGGGAAACCAACTATCGAGATTGTAT | for amplifying 5'-Gal 80 sequence |

^a^Restriction enzyme recognition sites are underlined.

**Fig. S1**


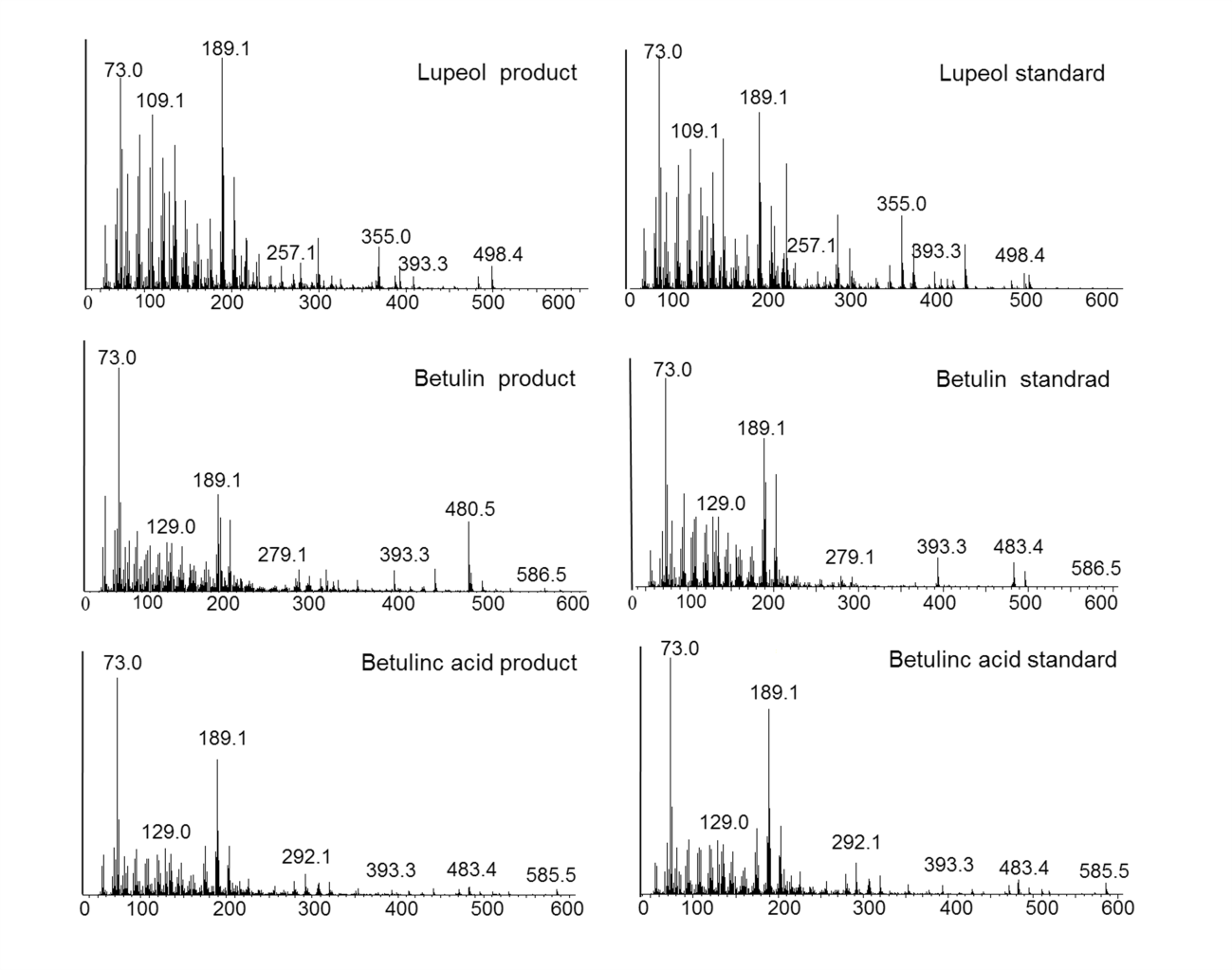


**Fig. S1** The mass fragmented patterns of the products lupeol (LUP), betulin (BN) and betulinic acid (BA) as well as their corresponding chemical standards.

**Fig. S2**

**
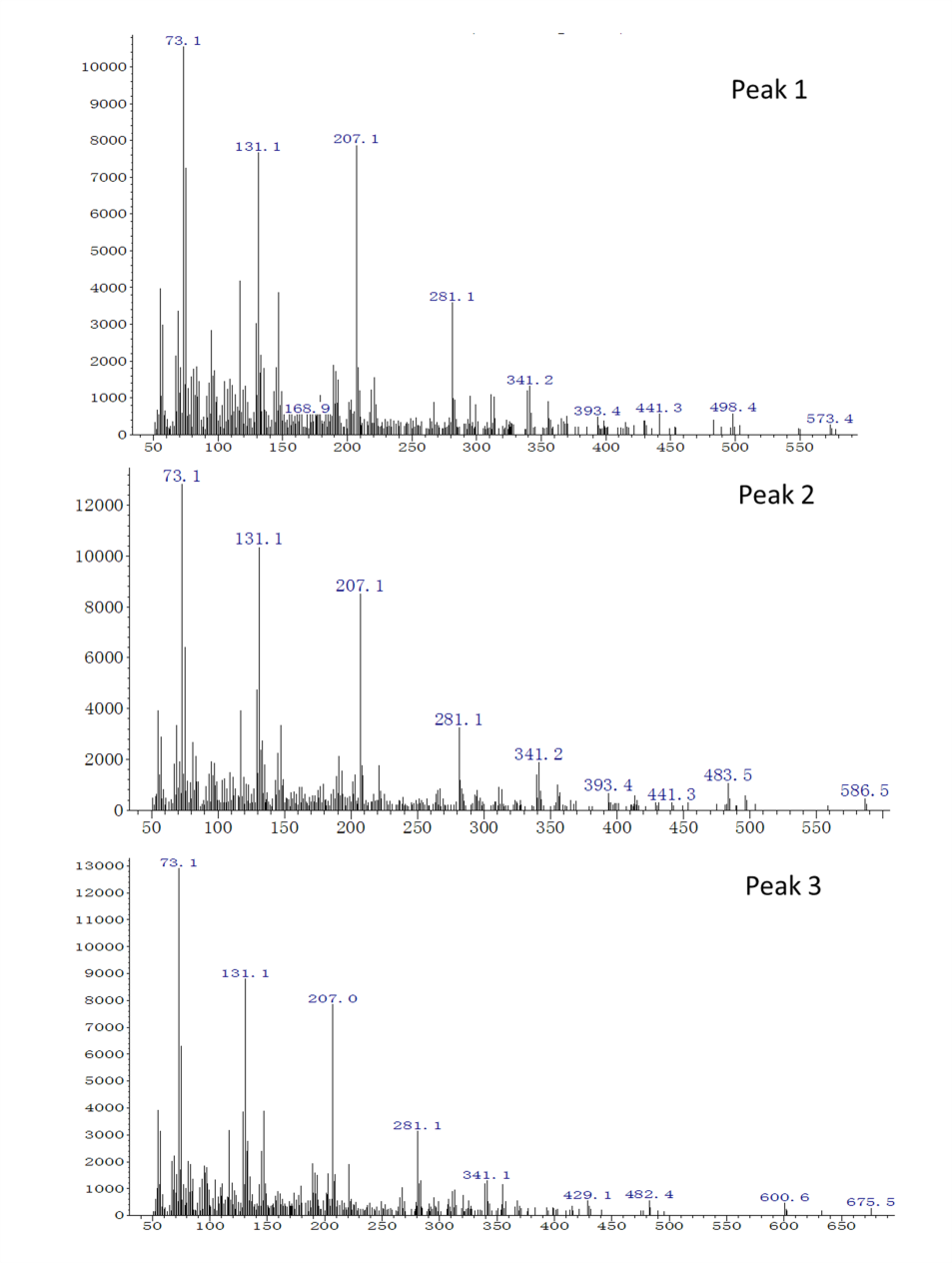
**

**Fig. S2** The MS spectrums of the unknown peaks 1-3 shown in the Fig. 1 of the main text.

**Fig. S3**

**
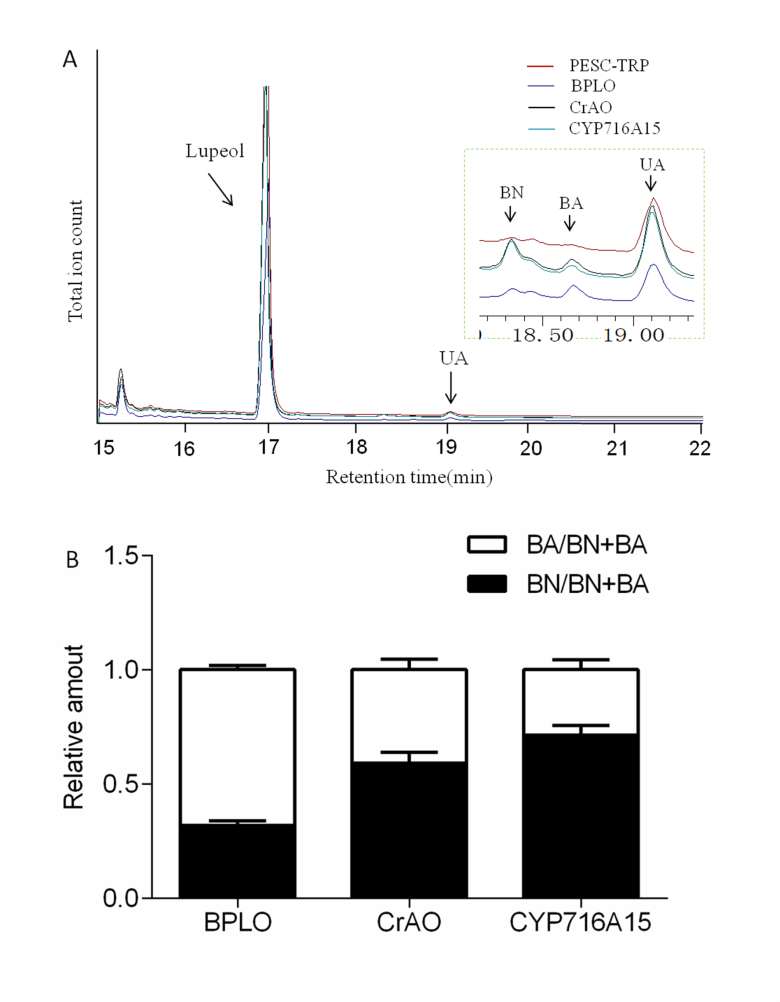
**

**Fig. S3** GC-MS analysis of the products extracted from the lupeol C28-oxidase (LO) alone-expressed yeast cultures fed with lupeol. Each LO enzyme (BPLO, CrAO and CYP716A15) was individually expressed in the WAT11 yeast strain, and resultant each transgenic yeast culture was then fed with lupeol at a final concentration of 50 µM upon the induction by 2% galactose. After the induction, the compounds were extracted from the mediums and cells, respectively. Trace amounts of betulin (BN) and betulinic acid (BA) were detected in the culture mediums while were almost undetectable in the cells. A, Total ion chromatograms were shown for the products by expressing BPLO (purple line), CrAO (black line), CYP716A15 (blue line) and the empty vector control (red line). The inset is the enlarged chromatograms from 17.5 to 19.5 minutes. B, the relative amounts of the products (BN and BA) produced by the transgenic yeast cells expressing the individual LO (BPLO, CrAO and CYP716A15). BN, betulin; BA, betulinic acid; UA, ursolic acid, which was used as an internal standard.

**Fig. S4**





**Fig. S4** The production of betulinic acid (BA) was mostly found in the yeast culture mediums of the both CEN.PK-ATR1-LB and WAT11-406-LB strains with relatively much less BA being detected inside their cells. Error bars represent the standard errors of the means calculated from three biological replicates.

**Fig. S5**


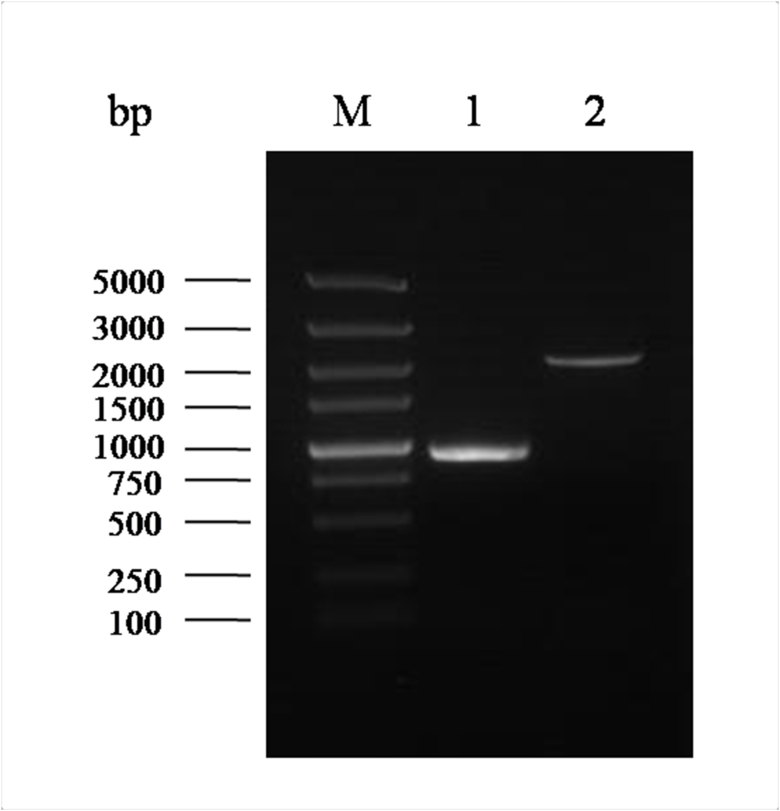


**Fig.** **S5** Confirmation of the Gal80p gene disruption by diagnostic PCRs. The PCRs were carried out using primers 23/24, which would yield the 897-bp amplified product for the wild type strain while 2094-bpamplified product for the Gal80p mutant strain. Lane 1, the amplified product using the genomic DNA of the wild strain WAT11-LB as the template; Lane 2, the amplified product using the genomic DNA of the Gal80p mutant strain as the template.

**Fig. S6**

**

**

**Fig. S6**  Comparison of the *BPLO* transcripts between the wild type strain WAT11-LB and the mutant WAT11-LB-△Gal80 under 2% galactose as the carbon source. The yeast cells collected at the static growth period were used for the gene expression analysis. Error bars represent the standard errors of the means calculated from three biological replicates.
